# Hypofractionated Radiotherapy (25 Gy/5) for Rare Superior Vena Cava Invasion by Papillary Thyroid Carcinoma: A Case Report

**DOI:** 10.7759/cureus.106301

**Published:** 2026-04-01

**Authors:** Ruijia Jin, Daegan Sit

**Affiliations:** 1 Radiation Oncology, University of British Columbia and BC Cancer, Vancouver, CAN

**Keywords:** external beam radiotherapy (ebrt), moderate hypofractionation, papillary carcinoma of thyroid, papillary carcinoma thyroid, superior vena cava (svc) obstruction, supportive and palliative care

## Abstract

Papillary thyroid carcinoma (PTC) typically carries an excellent prognosis with high survival rates and indolent behavior in most patients. Direct invasion or tumor thrombus extending into the great veins of the neck, including the superior vena cava (SVC), is exceptionally rare.

We present the case of an 84-year-old woman with PTC complicated by SVC invasion and partial luminal obstruction. She was diagnosed with pT4aN1 PTC, initially managed with thyroidectomy, two courses of I-131 therapy, neck dissection for residual disease, and adjuvant external beam radiotherapy (EBRT). This was followed by years of indolent disease until mediastinal nodal progression with SVC invasion and occlusion of the left subclavian and internal jugular veins. The patient declined surgery and endovascular intervention but accepted a short course of palliative EBRT with 25 Gy in 5 fractions.

Short-course palliative EBRT resulted in significant radiographic improvement over the course of a year without hemorrhagic complications.

This case provides novel information about the indolent natural history of PTC even with SVC invasion and the potential utility of moderately hypofractionated palliative EBRT in this setting.

## Introduction

Papillary thyroid carcinoma (PTC) is a well-differentiated thyroid cancer (WDTC) and the most common subtype of thyroid carcinoma, accounting for approximately 84% of cases [1]. It generally carries an excellent long-term prognosis, with 10-year survival rates exceeding 90% to 95% [1,2]. Only a small subset of patients (1.3%) develop locally advanced or high-risk disease, characterized by large tumor size, extrathyroidal extension, perineural invasion, nodal metastases, and positive surgical margins, all of which are associated with increased recurrence risk and worse outcomes [3,4]. Most oncological cases of SVC syndrome are attributed to lung cancer or lymphoma, and severe symptoms typically occur at 70%-90% obstruction of the superior vena cava (SVC) lumen [5-7]. Metastatic invasion of the SVC by WDTC is exceedingly rare, with only sporadic cases documented in the literature [8,9]. Management of WDTC with great vessel invasion poses significant challenges, as surgical resection may carry prohibitive operative risk and there is no established consensus on optimal management. The role of external beam radiotherapy (EBRT) in this context is poorly defined, with little to no literature on its efficacy and safety. 

## Case presentation

We present the case of a 77-year-old female who, at initial presentation in February 2017, identified a right-sided neck mass. She was otherwise asymptomatic. Her comorbidities included type 2 diabetes mellitus, hypertension, and gastroesophageal reflux. Her medications included metformin, quinapril/hydrochlorothiazide, levothyroxine, rabeprazole, and domperidone. She had no smoking, alcohol, or recreational drug use history, and she was well supported by her family. The patient had no known family history of thyroid or other malignancies and no known genetic syndromes.

To investigate the right neck mass, neck ultrasound demonstrated a 2 cm vascular nodule in the right thyroid lobe and a large echogenic, vascular, noncalcified mass in the left thyroid lobe. Fine-needle aspiration in May 2017 revealed papillary thyroid carcinoma.

She underwent total thyroidectomy in July 2017, which revealed bilateral advanced disease. On the right, there was direct invasion of the esophagus and lateral tracheal wall, necessitating the sacrifice of the right recurrent laryngeal nerve and part of the muscular wall of the esophagus. On the left, there was a direct invasion of the carotid sheath and the internal jugular vein. Pathology revealed multifocal bilateral PTC with a dominant 5cm lesion, associated with extrathyroidal extension and perineural invasion. Margins were positive. Three out of 12 removed lymph nodes were noted to be positive.

**Figure 1:**
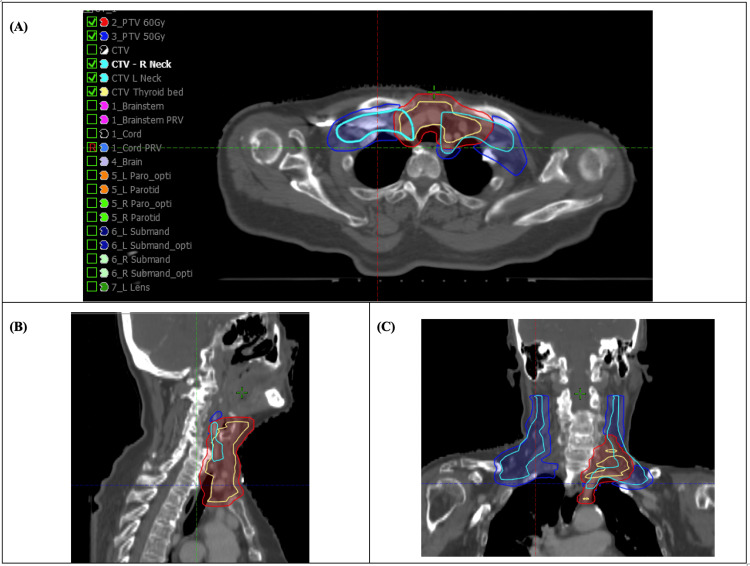
Adjuvant radiation target volumes for the thyroid bed and bilateral neck. Planning CT images demonstrating target volumes for adjuvant external beam radiation therapy (EBRT). Representative axial (A), sagittal (B), and coronal (C) views illustrate the prescribed dose distributions. The contours denote the 60 Gy planning target volume (PTV) for the postoperative thyroid bed and the 50 Gy PTV for the elective bilateral neck nodes. EBRT, external beam radiation therapy; PTV, planning target volume

Her case was reviewed at the Thyroid Multidisciplinary Conference in July 2017, where adjuvant high-dose radioactive iodine (RAI) followed by EBRT was recommended. She received 100 milliCurie (mCi) of I-131 in August 2017 with uptake seen in the neck. The dosing schedule was as follows: on days 1 and 2, 0.9 mg of Thyrogen (thyrotropin alfa) was delivered via intramuscular injection. On day 3, the RAI was delivered as a single dose. Residual disease was seen in the right supraclavicular nodes, and a second surgery by means of right neck dissection was performed in November 2017, yielding one additional positive lymph node. EBRT was then delivered: 60 Gy in 25 fractions to the postoperative thyroid bed and 50 Gy in 25 fractions to bilateral neck levels II-V elective nodes and level VI on the side of the primary [10]. The inferior extent of the planning target volume (PTV) was to the superior extent of the sternum. This was completed in February 2018. Axial, coronal, and sagittal snapshot images of her radiation plan can be found in Figures 1A-1C.

This was followed by a three-week supportive care admission to the hospital for radiation esophagitis.

In May 2018, surveillance CT raised concern for progressive lymph nodes in the upper mediastinum. Her case was again presented at the Thyroid Conference, and additional RAI was recommended. She received 150 mCi of I-131 in August 2018. Similar to her previous course, 0.9 mg Thyrogen was given intramuscularly on days 1 and 2, followed by the RAI on day 3 in a single dose. Over subsequent years, the patient underwent multiple rounds of surveillance imaging every 6 months (CT head, neck, and chest with contrast) with stable mediastinal findings, and she remained asymptomatic.

In March 2024, CT chest showed significant progression of abnormal soft tissue/conglomerate lymph nodes within the anterior mediastinum, notably invading the SVC with mild-to-moderate luminal narrowing. She was re-referred to Radiation Oncology. She was noted to be ECOG (Eastern Cooperative Oncology Group Performance Status) 3. Referral to thoracic surgery and interventional radiology or palliative EBRT was discussed, but the patient declined all interventions at that time. The patient was offered genetic testing and molecular profiling to explore the pathophysiology of the tumor and consider other systemic options; however, the patient declined these tests and did not wish to consider systemic therapy. In addition, the patient declined a fresh biopsy for personalized genomics as she felt it would be too invasive. She favored observation, given her asymptomatic status and prioritization of quality of life. Observation was deemed an acceptable option by multidisciplinary review, given her wishes, performance status, and the potential risk of hemorrhage from rapid tumor regression. This hemorrhagic risk was related to malignant lymph node invasion and erosion of the SVC wall, with potential for rapid tumor regression of the node leading to vessel wall disruption, resulting in massive hemorrhage and possible death.

**Figure 2:**
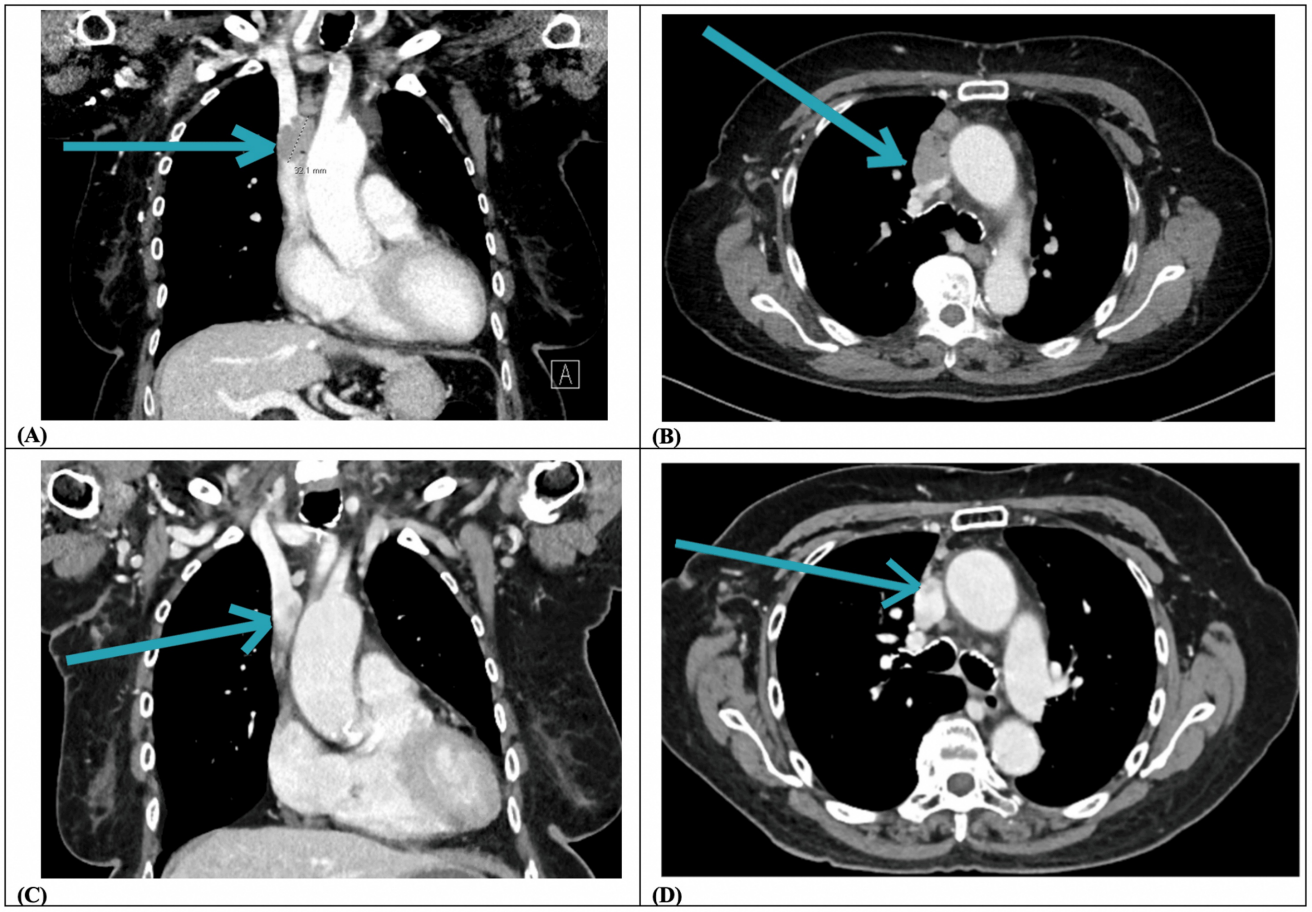
CT images before versus after EBRT. (A) June 2024: Coronal view of CT neck and chest with contrast showing 2/3 luminal occlusion of the SVC (blue arrow). (B) June 2024: Axial view of CT neck and chest with contrast showing the same finding (blue arrow) as in (A). (C) June 2025: Coronal view of CT neck and chest with contrast showing near-complete regression of the tumor thrombus within the SVC (blue arrow). (D) June 2025: Axial view of contrast-enhanced CT of the neck and chest showing the same finding (blue arrow) as in (C). EBRT, external beam radiotherapy

By June 2024, repeat CT showed further progression of the retrosternal lymphadenopathy measuring 4.4 cm (transverse) × 2.4 cm (anteroposterior) × 3.3 cm (craniocaudal). Most notably, there was a two-thirds luminal occlusion of the SVC, compared to a previously one-third occlusion (Figures 2A-2B). Occlusion of the medial left subclavian and lower left internal jugular veins was also noted. After discussion with Radiation Oncology and Thoracic Surgery, the patient again declined surgery or endovascular intervention.

Multidisciplinary Thyroid Conference recommended presenting EBRT as an option, given the degree of occlusion of the SVC and the increasing speed of progression in the SVC over the past year, which would likely become life-threatening given the pace of disease. RAI was not recommended as the macroscopic size (4.4 cm) of the tumor was thought to be too large to have a meaningful response. A variety of radiotherapy treatment options were considered, including a longer course of palliative radiotherapy (60-70 Gy in 30-35 fractions) versus shorter courses of 8 Gy in 1 fraction to 30 Gy in 10 fractions.

**Figure 3:**
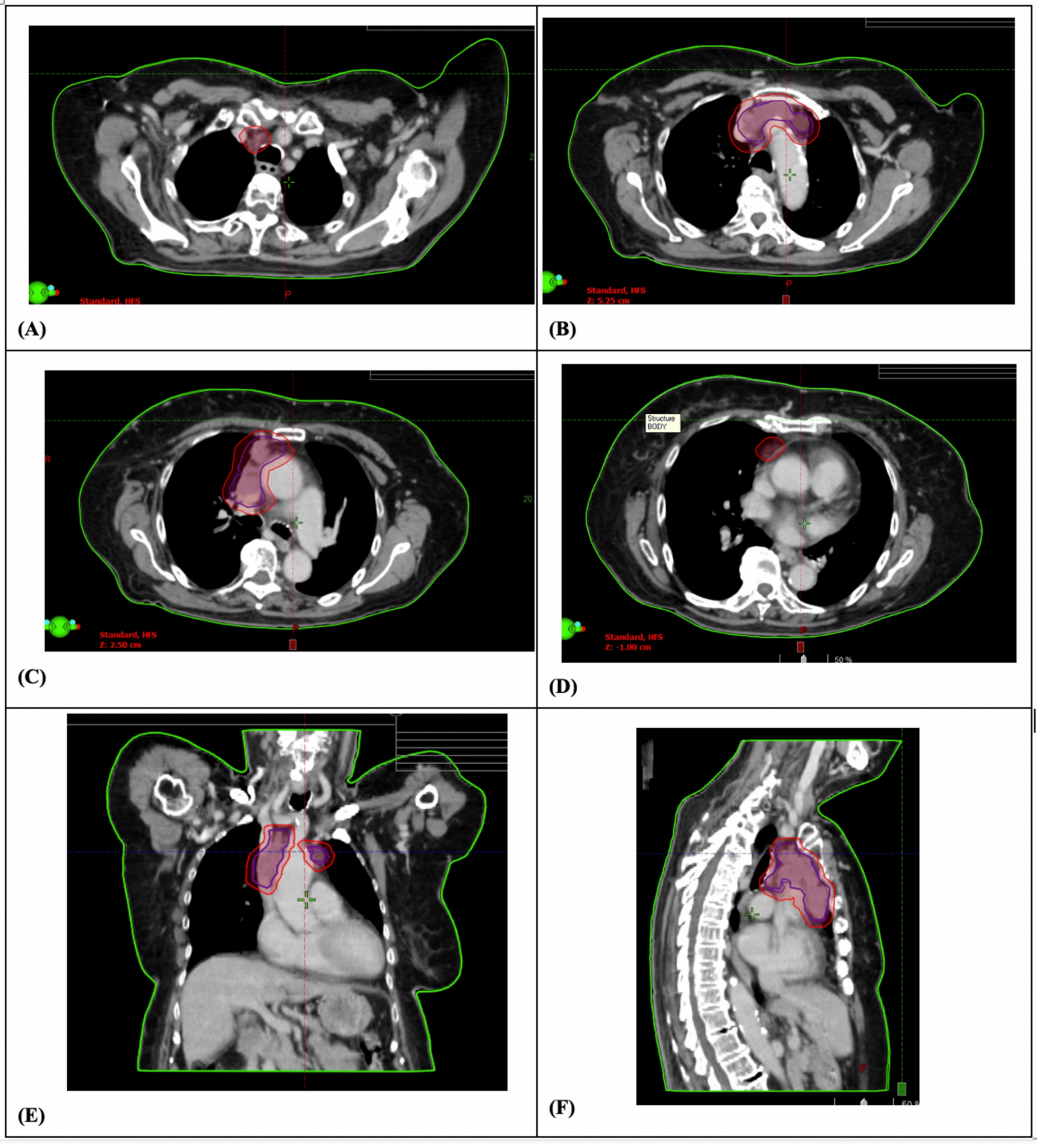
EBRT radiation target volumes to mediastinum and SVC nodes. Planning CT images demonstrating target volumes for EBRT to treat tumor thrombus invading the SVC. Representative axial images illustrate the superior (A), sequential posterior extents (B-D), and posterior-most extent (D) of the treatment volumes. Coronal (E) and sagittal (F) views further depict the spatial distribution of the treatment fields and dose coverage. EBRT, external beam radiation therapy; SVC, superior vena cava

Ultimately, through shared decision making, the patient consented to EBRT with 25 Gy in 5 fractions to the mediastinum and SVC nodes. She was treated with volumetric arc therapy (VMAT), and a 4D CT simulation scan was utilized to account for respiratory motion and maximize accuracy. The gross tumor volume of the SVC metastasis (GTVm) was contoured in all breathing positions and fused to ensure coverage of the metastasis (iGTVm), and a 5 mm PTV margin was used from the GTVm to account for intrafraction motion. The radiotherapy contours and plan were peer-reviewed with another staff radiation oncologist, and the radiotherapy was delivered via VMAT with 6 MV energy. Axial, coronal, and sagittal snapshot images of her radiation plan can be found in Figures 3A-3F.

She tolerated treatment without complications. The patient remained clinically well over the next year with no hemorrhagic complications or other toxicity related to this course of palliative EBRT. Follow-up imaging in June 2025 revealed near complete regression of mediastinal disease and thrombus within the SVC and left subclavian vein, with no new concerning findings. The tumor thrombus within the SVC was measured to be 0.6 cm craniocaudally, significantly decreased from its size of 3.3 cm before treatment (Figures 2C-2D).

## Discussion

Papillary thyroid carcinoma invading the SVC represents an exceptionally rare clinical scenario, with only sporadic cases reported in the literature [8,11]. Most cases of thyroid carcinoma-associated venous invasion involve the internal jugular or brachiocephalic veins; direct SVC involvement is particularly unusual. In the majority of published reports, management has centered on aggressive surgical resection with or without endovascular reconstruction, complemented by RAI and, occasionally, adjuvant EBRT or long courses of EBRT [8,11]. To our knowledge, there are no prior reports describing durable radiographic regression of SVC invasion from well-differentiated thyroid carcinoma (WDTC) treated solely with moderately hypofractionated EBRT (25 Gy in 5 fractions), highlighting the novelty of this case.

Surgical approaches for WDTC with SVC invasion

A 2002 study by Koike et al. described a 26-year-old woman with PTC extending from the brachiocephalic vein into the SVC. This patient underwent surgical resection with thrombectomy, along with postoperative RAI, and remained free of disease 8 months later [11]. This case highlights that aggressive surgical management can achieve a favorable outcome when the disease is anatomically resectable and the patient is an appropriate surgical candidate. A case series by Hyer et al. reports two patients with WDTC invading the SVC who were treated with a multimodal approach including surgery, RAI, and EBRT. One case was an 81-year-old woman with follicular carcinoma who underwent upfront thyroidectomy and resection of the right internal jugular vein. After surgery, she developed SVC syndrome due to venous thrombus occlusion from the tumor. She was treated with RAI and EBRT to the neck and superior mediastinum with a total dose of 60 Gy. She survived for over five years before succumbing to progressive metastatic disease. Another case involved a 70-year-old man with high-grade PTC significantly compressing the SVC due to bulky right hilar adenopathy. He underwent total thyroidectomy and neck dissection, followed by RAI. Within nine months, recurrence was seen, and he subsequently received a total of 50 Gy EBRT to the neck and mediastinum. He lived with locally controlled disease for 26 months [8]. This series highlights the complexity of PTC invasion into SVC with the potential benefit of a multimodality therapeutic approach.

Role of EBRT for WDTC with SVC invasion

Historically, PTC and WDTC are considered less radiosensitive to EBRT, with most evidence indicating limited efficacy for locoregional control. PTC is characterized by a low mitotic rate and lack of necrosis, which results in its indolent clinical behavior and generally excellent prognosis. These features, however, also correlate with slow growth and limited responsiveness to cytotoxic therapies such as EBRT [12]. The American Head and Neck Society specifically states that WDTC generally does not respond robustly to EBRT, and its use should be highly selective [13]. EBRT can be considered to improve locoregional control in patients with gross residual or unresectable disease, particularly in older patients or those with extensive extrathyroidal invasion [14,15]. In the metastatic setting, the latest guidelines from the American Thyroid Association state EBRT can still be considered for gross residual or unresectable disease, but the impact of locoregional disease progression must be weighed in the context of overall prognosis in patients with distant metastases. This is especially important in the absence of data showing improvement in overall survival and known risks of acute and long-term treatment-related toxicity. Local therapies, including radiotherapy, should, however, be considered for symptomatic or anatomically threatening lesions with palliative intent [16].

The management of WDTC causing obstruction of great veins, such as the SVC, with EBRT (without surgery or stenting) is an exceptionally rare strategy. One 1991 case report by Wilford et al. presents an 86-year-old woman with follicular thyroid carcinoma progressing to SVC syndrome despite multiple doses of RAI. A total of 53 Gy of EBRT was delivered to the neck and upper mediastinum. This resulted in a significant and rapid reduction in the tumor mass as well as her symptoms of facial and neck edema. She was clinically well with no recurrence over four years. After the four-year mark, she developed a recurrence that required a tracheostomy and a second course of EBRT (26 Gy to the neck), which resulted in tumor reduction and symptom improvement [17]. Another case report by Demirer et al. describes a 52-year-old woman with inoperable follicular thyroid carcinoma treated with 50 Gy in 25 fractions to the thyroid bed and gross disease. The tumor regressed remarkably in six months on imaging [18].

Radiobiological rationale for 25 Gy in 5 fractions

When discussing dose and fractionation for EBRT to PTC, the American Head and Neck Society recommends higher doses (≥60 Gy) in definitive or adjuvant settings in select patients with unresectable or gross residual disease [13]. For palliative thyroid carcinoma cases, the most common dose and fractionation schedules are 20 Gy in 5 fractions or 30 Gy in 10 fractions [19]. EBRT is a recognized palliative intervention for malignant SVC obstruction, particularly when rapid symptom relief is required, and other modalities such as surgery or endovascular intervention are not feasible or declined [20]. Twenty Gy in 5 fractions and 30 Gy in 10 fractions are typical palliative EBRT regimens for SVC obstruction [21].

In our case, following a shared decision-making process, a radiation regimen of 25 Gy in 5 fractions was selected to balance treatment efficacy with the risk of catastrophic hemorrhage from rapid tumor regression. A 25 Gy in five fractions regimen delivers a biologically effective dose (BED) of 37.5 Gy (using an alpha-beta ratio of 10). In comparison, standard palliative regimens of 20 Gy in 5 fractions deliver a BED of 28 Gy, while 30 Gy in 10 fractions delivers a BED of 39 Gy (using the same alpha-beta ratio of 10). While the BED values for the 25 and 30 Gy regimens are similar, 25 Gy in 5 fractions was favored to minimize treatment duration, given the patient’s performance status and her preference to reduce the number of appointments. Longer radiation courses, such as 50-70 Gy in 25-35 fractions (BED of 60-84 Gy), were considered but deemed inappropriate due to the patient's performance status and potential toxicities.

Our patient tolerated this treatment without acute complications, and follow-up imaging confirmed significant radiographic improvement in thrombus size. She remained clinically well with no hemorrhagic complications in the 12 months following EBRT. A detailed summary and timeline of her clinical case are provided in Table 1.

**Table 1:** Summary and timeline of case presentation. PTC, papillary thyroid carcinoma; IJV, internal jugular vein; RAI, radioactive iodine; EBRT, external beam radiotherapy; mCi, milliCurie; I-131, iodine-131; CT, computed tomography; SVC, superior vena cava; IR, interventional radiology; MTC, multidisciplinary thyroid conference; BED, biologically effective dose; VMAT, volumetric modulated arc therapy; iGTV, internal gross target volume; PTV, planning target volume; MV, megavoltage

Date	Event	Details and findings
February 2017	Initial presentation	The patient presented with a right neck mass. Neck ultrasound showed a 2 cm vascular nodule (right thyroid lobe) and a large echogenic vascular mass (left thyroid lobe).
May 2017	Fine-needle aspiration	Papillary thyroid carcinoma (PTC) confirmed.
July 2017	Total thyroidectomy	Bilateral advanced disease was seen. - Right: invasion of esophagus and trachea → this led to sacrifice of right recurrent laryngeal nerve + esophageal wall resection. - Left: invasion of the carotid sheath and internal jugular vein (IJV). - Pathology: multifocal bilateral PTC (dominant 5 cm), extrathyroidal extension, perineural invasion, positive margins, 3/12 nodes positive
July 2017	Multidisciplinary Thyroid Conference	The conference recommended adjuvant high-dose radioactive iodine (RAI) followed by external beam radiotherapy (EBRT).
August 2017	Adjuvant RAI	100 mCi I-131 administered; uptake seen in the neck. Residual disease in right supraclavicular nodes.
November 2017	Right neck dissection	Performed for residual disease in right supraclavicular nodes; 1 positive lymph node found.
February 2018	Adjuvant EBRT	60 Gy in 25 fractions delivered to the thyroid bed and bilateral neck, followed by a three-week hospital admission for supportive care
May 2018	Disease progression on surveillance CT	The CT scan showed progressive upper mediastinal lymph nodes.
August 2018	Additional RAI	150 mCi I-131 administered. Stable disease afterward; asymptomatic for several years.
March 2024	Disease progression on surveillance CT	The CT scan showed progression in the anterior mediastinum; SVC invasion with mild-moderate narrowing.
March 2024	Observation chosen	The patient declined interventions (surgery, IR, EBRT). Observation chosen due to asymptomatic status and hemorrhagic risk from rapid regression.
June 2024	Further disease progression	The CT scan showed 2/3 SVC luminal occlusion (previously 1/3); chronic occlusion of the medial left subclavian and lower left IJV.
June 2024	Multidisciplinary Thyroid Conference	The patient declined surgery or endovascular intervention again. Case reviewed again at MTC. EBRT recommended (given rapid SVC progression). RAI not advised (tumor too large). Options discussed: 60–70 Gy/30–35#, 30 Gy/10#, or 8 Gy/1#.
July 2024	Higher biologically effective dose (BED) regimen of palliative EBRT	25 Gy in 5 fractions delivered to mediastinum and SVC nodes. VMAT technique with 4D CT sim, iGTVm, and 5 mm PTV margin. Peer-reviewed plan, 6 MV energy used. No complications occurred.
June 2025	Clinical and radiographic follow-up	The patient was seen clinically and was well, with no toxicity or hemorrhagic events. CT scan showing near-complete regression of mediastinal disease and SVC/subclavian thrombus; no new concerning findings.

## Conclusions

EBRT with 25 Gy in 5 fractions to WDTC invading into the SVC resulted in a complete response of tumor, preservation of our patient’s performance status, and no major complications in the year following treatment. This case offers several unique insights. The first is that WDTC can rarely present with aggressive vascular invasion despite an otherwise indolent course. Another highlight is patient-centered management aligned with individual goals of care. In an elderly patient who prioritizes quality of life, aggressive surgical or endovascular intervention may reasonably be avoided. Finally, although PTC is generally considered relatively radioresistant, moderately hypofractionated EBRT with 25 Gy in 5 fractions can achieve meaningful local control and significant radiographic response without toxicity. While the near-complete regression at 12 months is encouraging, the long-term durability of this response remains to be established. Ongoing clinical surveillance, including routine follow-up and CT scans, will be essential to monitor for late radiation-induced toxicities and ensure early detection of any eventual local recurrence. Further research is needed to define if and when EBRT is an appropriate management strategy for locally advanced WDTC with aggressive vascular invasion. Additionally, further investigation is warranted to clarify the radiosensitivity of PTC in the setting of unresectable disease. This may help identify patient and tumor factors that predict EBRT response and toxicity, along with integration with other modalities.
